# Supplementary material for: Enhancing Conformational Sampling for Intrinsically Disordered and Ordered Proteins by Variational Autoencoder
Source: Int J Mol Sci. 2023 Apr 7;24(8):6896. doi: 10.3390/ijms24086896 (PMC10138423; doi:10.3390/ijms24086896)
Supplement: Supplementary file 1 [file ijms-24-06896-s001.zip › ijms-2228962-supplementary.pdf]

Supporting Information

Enhancing Conformational Sampling for Intrinsically Disordered and Ordered Proteins by Variational Autoencoder

Jun-Jie Zhu [1,†], Ning-Jie Zhang [1,†], Ting Wei [1] and Hai-Feng Chen [1,2,*]

[1] State Key Laboratory of Microbial Metabolism, Joint International Research Laboratory of Metabolic & Developmental Sciences, Department of Bioinformatics and Biostatistics, National Experimental Teaching Center for Life Sciences and Biotechnology, School of Life Sciences and Biotechnology, Shanghai Jiao Tong University, Shanghai 200240, China
[2] Shanghai Center for Bioinformation Technology, Shanghai 200240, China
[*] Correspondence: haifengchen@sjtu.edu.cn; Tel./Fax: +86-21-34204073
[†] These authors contributed equally to this work.

Table S1. Experimental chemical shifts used in this work.

Protein Systems	Experimental Measurements	Data sources
	Intrinsically Disordered Proteins	
RS1		[1]
Aβ40		[2]
PaaA2	Backbone Cα chemical shifts	[3]
R17		
α-synuclein		[4]
	Structural Proteins	
Ubiquitin	Backbone Cα chemical shifts	[5]
BPTI		[6]

Table S2. Experimental Rg used in this work.

Protein Systems	Experimental Rg value	Data sources
RS1	12.62 ± 0.07	[7]
Aβ40	12.0 ± 1.3	[8]
PaaA2	22.4 ± 4.0	[3]
R17	22.9 ± 1.1	[9]
α-synuclein	31.5 ± 5	[10, 11]

Table S3. Structure of VAEs of different layers. The latent space is set to a dimension of 2. The number of neurons in input and output layers is $3N_{Atom}$, i.e. the cartesian coordinates of all input atoms.

Hidden layers	Encoder	Latent space	Decoder
**1**	128		128
**2**	512, 32		32, 512
**3**	1024, 128, 16	2	16, 128, 1024
**4**	1024, 256, 64, 16		16, 64, 256, 1024
**5**	2048, 512, 128, 32, 8		8, 32, 128, 512, 2048

Table S4. Model training and conformation generating speed of AE and VAE, compared to MD simulation.

Method	Time for training 100 epochs(s)	Time for generating 5W conformations (s)
**AE**	568.88	6.97
**VAE**	402.04	5.49
**MD**	$\sim 140\ hours$	

Table S5. Properties of IDP systems used in this work.

Protein Systems	Structural properties
RS1	Fully extended, arginine residues in charged states. [7]
Aβ40	Partly folded, α-helical conformations favored. [8]
PaaA2	Compact, two preformed helices, connected by a flexible linker. [3]
R17	Foldable but naturally extended, highly hydrophilic. [9]
α-synuclein	Lacks organized structure, random coil in dilute solution. [10, 11]

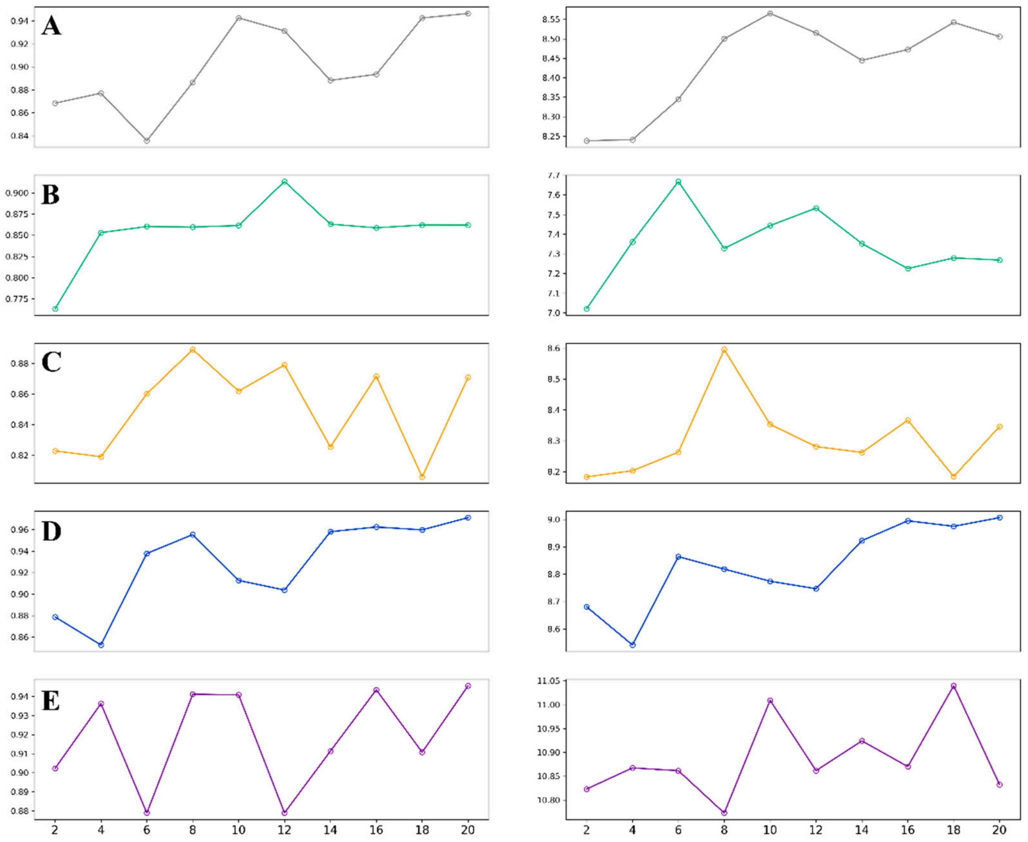

Figure S1. Mean spearman coefficient (left) and RMSD (right) recorded in different epochs. (A) RS1, (B) Abeta40, (C) PaaA2, (D) R17, (E) α-synuclein.

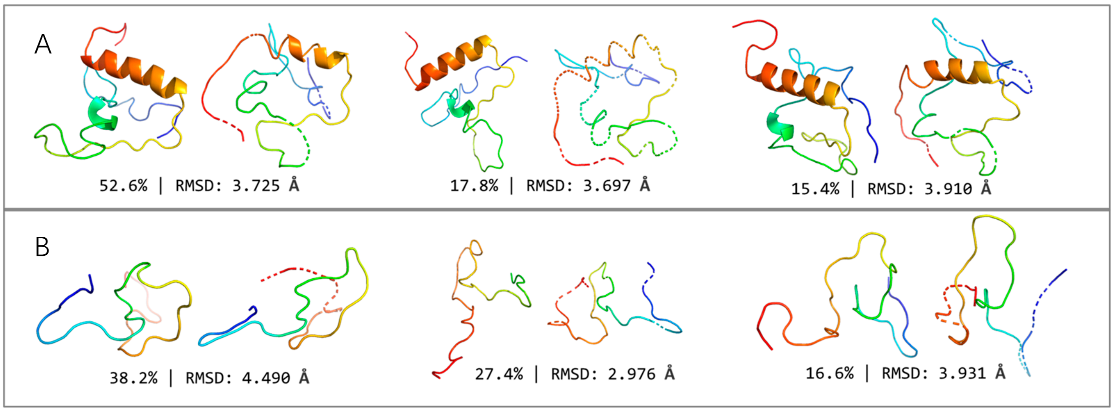

Figure S2. Generated structures that are centroids of the top 3 clusters (right) compared to conformations in MD trajectories (left). (A) PaaA2, (B) Abeta40.

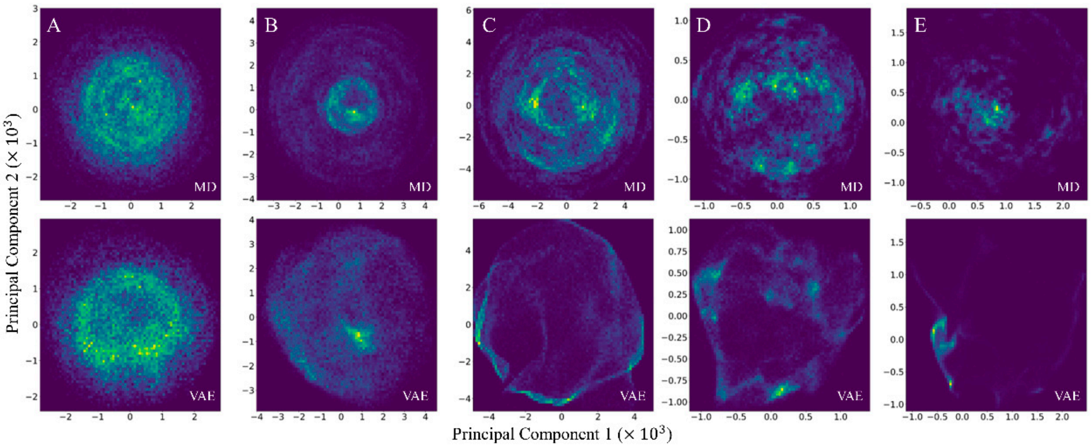

Figure S3. Principle component reduction of MD trajectories and generated IDP ensembles. (A) RS1. (B) Abeta40. (C) PaaA2. (D) R17. (E) α-synuclein.

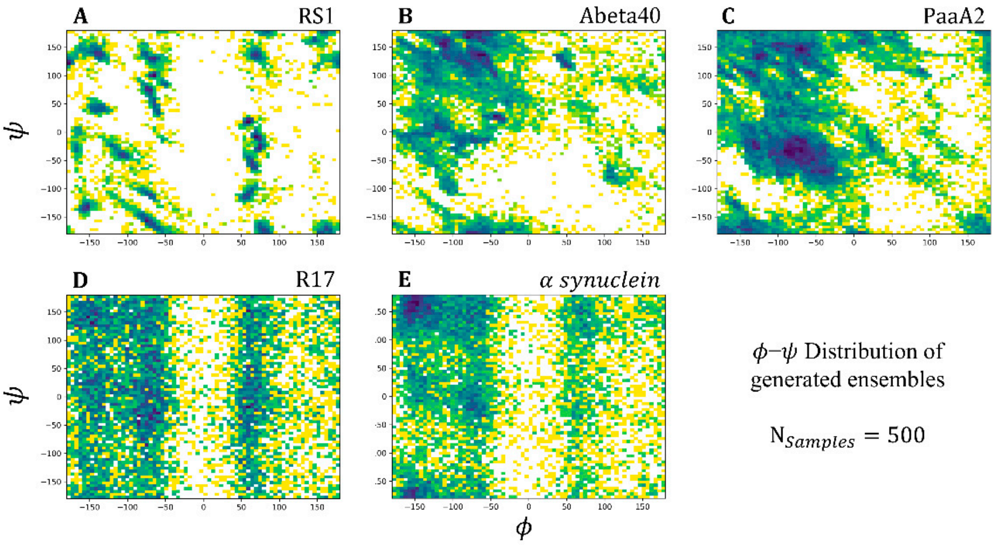

Figure S4. Ramachandran plots of MD trajectories and generated IDP ensembles. (A) RS1. (B) Abeta40. (C) PaaA2. (D) R17. (E) α-synuclein.

References

1. Cragnell, C., et al., *Coarse-grained modeling of the intrinsically disordered protein Histatin 5 in solution: Monte Carlo simulations in combination with SAXS.* (1097-0134 (Electronic)).
2. Hou, L., et al., *Solution NMR Studies of the Aβ(1−40) and Aβ(1−42) Peptides Establish that the Met35 Oxidation State Affects the Mechanism of Amyloid Formation.* Journal of the American Chemical Society, 2004. **126**(7): p. 1992-2005.
3. Sterckx, Yann G.J., et al., *Small-Angle X-Ray Scattering- and Nuclear Magnetic Resonance-Derived Conformational Ensemble of the Highly Flexible Antitoxin PaaA2.* Structure, 2014. **22**(6): p. 854-865.
4. Kang, L., et al., *Mechanistic insight into the relationship between N-terminal acetylation of α-synuclein and fibril formation rates by NMR and fluorescence.* (1932-6203 (Electronic)).
5. Cornilescu, G., et al., *Validation of Protein Structure from Anisotropic Carbonyl Chemical Shifts in a Dilute Liquid Crystalline Phase.* Journal of the American Chemical Society, 1998. **120**: p. 6836-6837.
6. Otting G.; Liepinsh E.; Wüthrich K. Disulfide bond isomerization in BPTI and BPTI(G36S): An NMR study of correlated mobility in proteins. *Biochemistry* **1993**, *32*, 3571. https://doi.org/10.1021/bi00065a008.
7. Rauscher, S., et al., *Structural Ensembles of Intrinsically Disordered Proteins Depend Strongly on Force Field: A Comparison to Experiment.* J Chem Theory Comput, 2015. **11**(11): p. 5513-24.
8. Granata, D., et al., *The inverted free energy landscape of an intrinsically disordered peptide by simulations and experiments.* Sci Rep, 2015. **5**: p. 15449.

9. Hofmann, H., et al., *Polymer scaling laws of unfolded and intrinsically disordered proteins quantified with single-molecule spectroscopy.* Proc Natl Acad Sci U S A, 2012. **109**(40): p. 16155-60.
10. Morar, A.S., et al., *Solvent-induced collapse of alpha-synuclein and acid-denatured cytochrome c.* Protein Sci, 2001. **10**(11): p. 2195-9.
11. Schwalbe, M., et al., *Predictive atomic resolution descriptions of intrinsically disordered hTau40 and α-synuclein in solution from NMR and small angle scattering.* Structure, 2014. **22**(2): p. 238-49.
